# Genomic and phenotypic analysis of a novel clinical isolate of *Corynebacterium pyruviciproducens*

**DOI:** 10.1186/s12866-023-03075-6

**Published:** 2023-12-06

**Authors:** Jiaqi Wang, Jiajia Feng, Wei Jia, Tingxun Yuan, Xinyu He, Qianqian Wu, Fujun Peng, Wei Gao, Zhongfa Yang, Yuanyong Tao, Qian Li

**Affiliations:** 1 School of Medical Laboratory, Weifang Medical University, Weifang, Shandong 261053 PR China; 2 Engineering Research Institute of Precision Medicine Innovation and Transformation of Infections Diseases, Weifang Medical University, Weifang, Shandong 261053 PR China; 3 Clinical Laboratory, Weifang Maternal and Child Health Care Hospital, Weifang, Shandong 261011 PR China; 4 Clinical Laboratory, Weifang People’s Hospital, Weifang, Shandong 261000 PR China; 5 Clinical Laboratory, the Affiliated Hospital of Weifang Medical University, Weifang, 261031 PR China; 6 School of Basic Medical Sciences, Weifang Medical University, Weifang, China; 7 Key Lab for Immunology in Universities of Shandong Province, Weifang Medical University, Weifang, Shandong 261053 PR China

**Keywords:** *Corynebacterium pyruviciproducens*, Antibiotic resistance, Virulence, Genomic analysis

## Abstract

**Background:**

*Corynebacterium pyruviciproducens* is a recently described species of *Corynebacterium*. There are few reports on the microbiological characteristics of the new species, and there is a lack of reports on the genomic analysis of the species.

**Results:**

This study involved a clinical isolate from the pus of a hospital patient with sebaceous gland abscesses. The clinically isolated strain was identified as *C. pyruviciproducens* strain WYJY-01. In this study, referring to Koch’s postulates, we observed the pathological changes of animal models infected by intraperitoneal injection and subcutaneous injection of pure culture of the strain WYJY-01. Furthermore, the strain WYJY-01 was isolated and cultured again from animal models' subcutaneous abscess drainage fluid. Subsequently, the genomics of the strain WYJY-01 was analyzed. By comparing various gene databases, this study predicted the core secondary metabolite gene cluster of the strain WYJY-01, virulence factor genes carried by prophage, pathogenicity islands, and resistance islands. In addition, the genomes of *C. pyruviciproducens* strain WYJY-01, ATCC BAA-1742^ T^, and UMB0763 were analyzed by comparative genomics, and the differential genes of strain WYJY-01 were compared, and their functions were analyzed.

**Conclusion:**

The findings showed that the strain WYJY-01 had pathogenicity, supplementing the phenotype characteristics of *C. pyruviciproducens*. Meanwhile, this research revealed the possible molecular mechanism of the pathogenicity of the strain WYJY-01 at the gene level through whole genome sequence analysis, providing a molecular basis for further research.

**Supplementary Information:**

The online version contains supplementary material available at 10.1186/s12866-023-03075-6.

## Background

*Corynebacterium* is a group of Gram-positive bacilli characterized by rod-like expansion at one or both ends, belonging to actinomycetes. *Corynebacterium* is mainly isolated from human, animal, soil, water, and other environmental resources [1, 2], most of which belong to normal flora, and some belong to conditional pathogens. Only a few bacteria in *Corynebacterium* are pathogenic to human beings [3], among which *Corynebacterium diphtheria* is the most specific pathogenic bacterium, and the primary pathogenic substance is diphtheria toxin, which is the expression product of lysogenic infection of β-bacteriophage [4]. Therefore, the prophage in the genetic material of *Corynebacterium* has a particular influence on the pathogenicity of *Corynebacterium*. In addition, *Corynebacterium* grows slowly in various media and needs some particular nutrients for its metabolic growth [5]. Among them, lipophilic *Corynebacterium* requires special culture conditions, which are difficult to isolate and culture in the routine clinical laboratory, and easy to miss diagnosis and misdiagnosis [6]. It is worth noting that many studies have shown that the main pathogenic bacteria of granulomatous mastitis are *Corynebacterium* [7]. However, the pathogenesis of the disease has not been studied. The researchers found preliminarily that most of the pathogenic *Corynebacterium* in this disease were lipophilic bacteria, and there were lipid bubbles around the bacteria in the abscess [8]. Meanwhile, *C. pyruviciproducens* has been found in the gut microbiomes of humans [9]. Therefore, the pathogenicity of lipophilic *Corynebacterium* has been widely discussed.

*Corynebacterium pyruviciproducens* is a recently described species of *Corynebacterium*. It was isolated from the inguinal abscess specimen collected by the American Medical Research Center in 2006 by Tong J, and was officially named in 2010 [10]. *C. pyruviciproducens* does not contain diphtheria toxin, tuberculous stearic acid, and other known virulence factors, and can be used as an immunomodulator to promote the host's humoral immune response to pathogenic microorganisms by regulating the function of macrophages [11, 12]. Then, D. Goldenberger et al. cultured urine, blood culture, skin infection, pus, joint cavity effusion, and other specimens from Switzerland and Canada and isolated *C. pyruviciproducens*. These clinical isolates of *C. pyruviciproducens* were found to differ from the type strain ATCC BAA-1742^ T^ in biochemical reaction and antimicrobial susceptibility [13]. As *C. pyruviciproducens* was discovered recently, there are relatively few literature reports related to this species, and there is a lack of research on its genomic analysis. Futhermore, up to present, there is an absence of studies describing the putative pathogenicity of this species. Therefore, the purpose of this research project is to supplement the microbiological characteristics of the pathogenicity of *C. pyruviciproducens*, and predict the molecular biological basis related to its microbiological characteristics through whole-genome sequence analysis.

## Results

### General microbiological characterization of *C*. *pyruviciproducens* strain WYJY-01

**Fig. 1:**
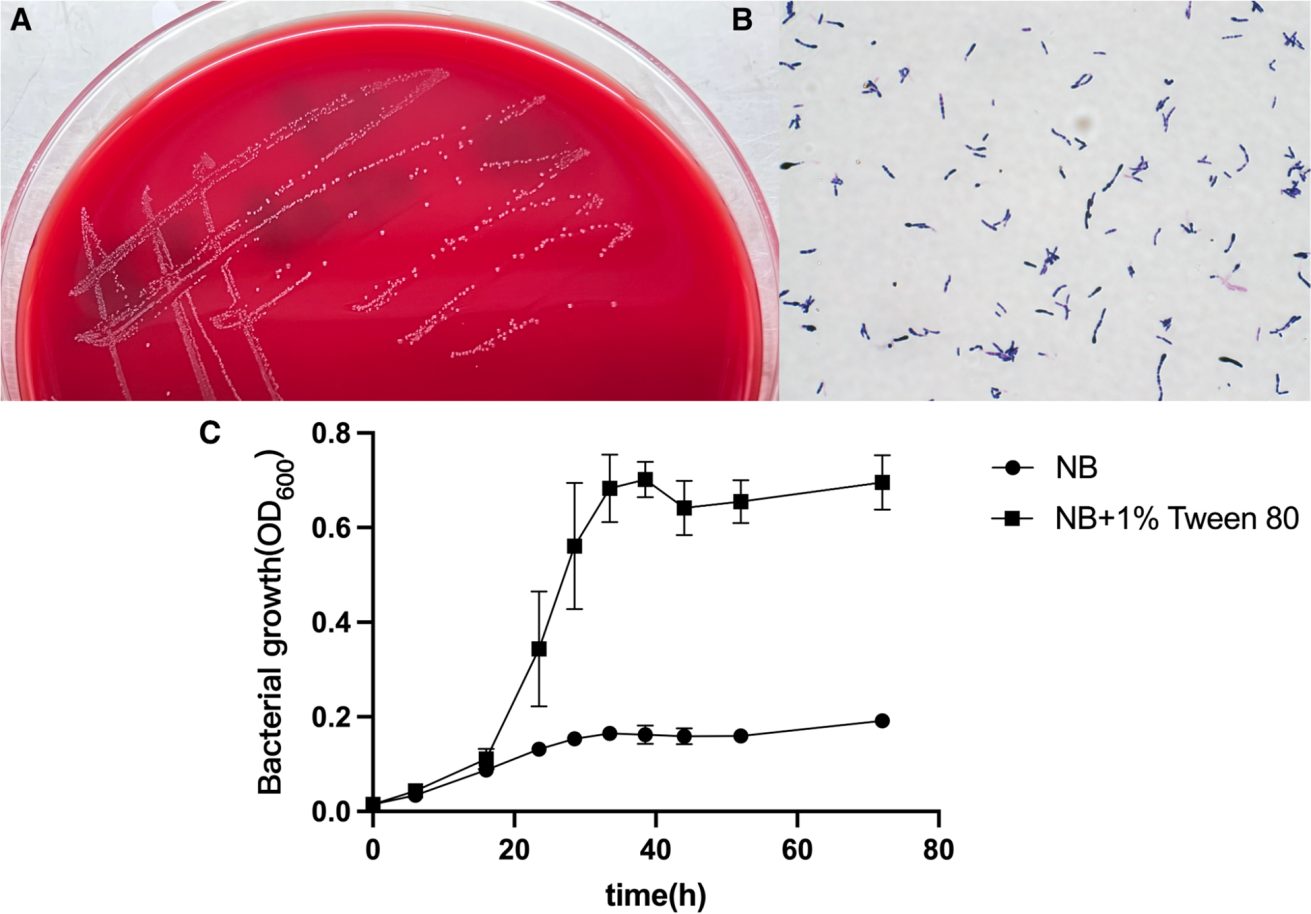
General identification of the clinical isolate of *C. pyruviciproducens* strain WYJY-01. **A** Colony characteristics of the strain WYJY-01 cultured on Columbia blood agar plates. **B** Morphological characteristics of the strain WYJY-01 under the light microscope after Gram staining. **C** Lipid requirement test and growth curve determination of the strain WYJY-01

*C. pyruviciproducens* strain WYJY-01 isolated and cultured from abscess drainage fluid was cultured on Columbia blood plates at 37 °C for 48 h, and its colony morphology was round and smooth, and the colony center was convex and transparent (Fig. 1A). The strain WYJY-01 was Gram-positive under the light microscope after Gram staining, and its shape was rod-shaped (Fig. 1B). However, the strain WYJY-01 was identified as *Kocuria rosea* by VITEK 2 Compact full-automatic microbial analyzer. Because *Kocuria rosea* is the Gram-positive cocci, it was evident that the results were not consistent with each other [14].

**Fig. 2:**
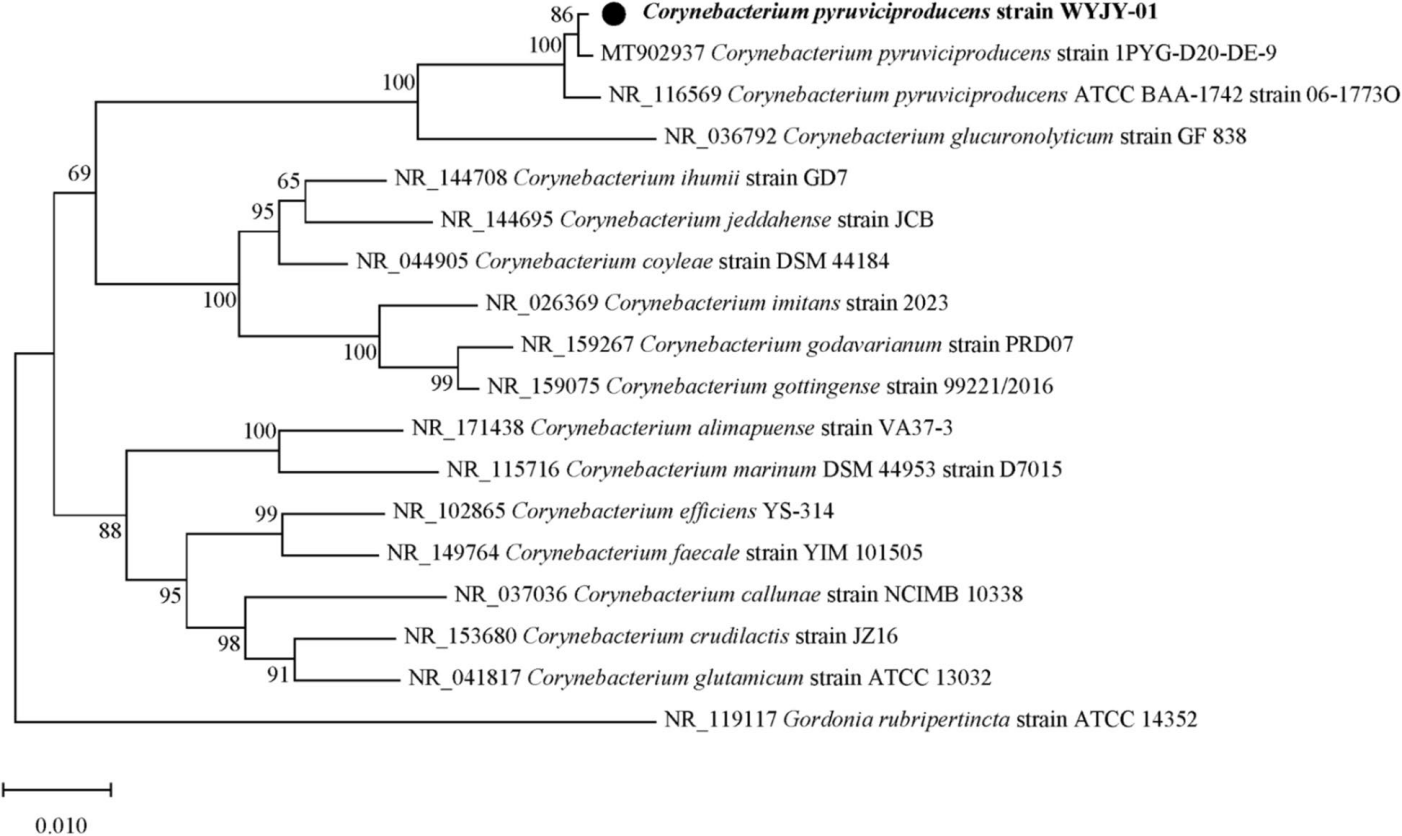
The phylogenetic tree of C. *pyruviciproducens* strain WYJY-01 obtained by the Neighbor-Joining method based on the 1393 bp 16S rRNA gene sequences. This tree shows the phylogenetic relationships between the strain WYJY-01 and closely related species

The comparison results based on the GenBank database showed that the 16S rRNA gene sequence of the strain WYJY-01 was the closest to *Corynebacterium sp.* 718260/2011 (GenBank JX501770.1), with a sequence similarity of 100%. The *rpoB* gene sequence of the strain WYJY-01 was the closest to that of *Corynebacterium sp.* NML00-0179 (GenBank GU304660.1), with a sequence similarity of 99.75%. These two strains were clinical isolates of *C. pyruviciproducens*. Subsequently, a phylogenetic analysis of the 16S rRNA gene with a tree constructed based on the Neighbor-Joining method showed that the strain WYJY-01 was a member of the genus *Corynebacterium* (Fig. 2).

The difference in biochemical characteristics between the strain WYJY-01 and the strain ATCC BAA-1742^ T^ was (Additional file 1: Table S1): CAMP reaction and reduction of nitrates of strain WYJY-01 are positive; fermentation of D-ribose, D-xylose, D-glucose, maltose and sucrose of strain WYJY-01 were negative.

According to the Clinical and Laboratory Standards Institute (CLSI) guidelines [15], the drug sensitivity of the strain WYJY-01 was measured by the Etest method (Table 1). The strain WYJY-01 was resistant to ceftriaxone, gentamicin, erythromycin, ciprofloxacin, and clindamycin. However, the strain ATCC BAA-1742^ T^ was sensitive to gentamicin and ciprofloxacin.

**Table 1:** Antimicrobial susceptibility testing results

Antimicrobial class	Antimicrobial agent^a^	Result for the strain ATCC BAA-1742^ T^		Result for the strain WYJY-01	
		MIC (μg·mL^−1^)	Interpretation^b^	MIC (μg·mL^−1^)	Interpretation
Penicillins	Penicillin	0.094	S	2	I
Penicillins	Ampicillin	ND^c^		0.5	NB
Penicillins	Amoxicillin-clavulanic acid	0.094	NB	ND	
Penicillins	Piperacillin-tazobactam	0.5	S	ND	
Cephems	Cefotaxime	ND		0.5	S
Cephems	Ceftriaxone	1.5	I	4	R
Cephems	Cefuroxime	0.125	NB	ND	
Cephems	Cefepime	0.25	S	ND	
Carbapenems	Imipenem	0.064	S	0.25	S
Glycopeptides	Vancomycin	0.38	S	1	S
Lipopeptides	Daptomycin	0.032	S	ND	
Aminoglycosides	Gentamicin	0.25	S	> 256	R
Macrolides	Erythromycin	2	R	> 256	R
Quinolones	Levofloxacin	ND		> 32	NB
Quinolones	Ciprofloxacin	0.19	S	> 32	R
Quinolones	Moxifloxacin	0.064	NB	ND	
Tetracyclines	Tetracycline	0.75	S	0.5	S
Tetracyclines	Tigecycline	0.032	NB	ND	
Lincosamides	Clindamycin	> 256	R	> 256	R
Folate pathway inhibitors	Trimethoprim-sulfamethoxazole	ND		1	S
Ansamycins	Rifampin	< 0.002	S	0.125	S
Oxazolidinones	Linezolid	0.38	S	0.25	S
Oxazolidinones	Linezolid	0.38	S	0.125	S

^a^The antimicrobial agents shown were tested by both laboratories either by Etest

^b^*S* sensitive, *I* intermediate, *R* resistant (or immune), *NB* no breakpoints by CLSI

^c^*ND* not done

*C. pyruviciproducens* strain WYJY-01 was cultured at 37 °C in nutrient broth (NB) medium and NB medium with 1% Tween 80 added. The OD_600_ of bacterial liquid in different media was measured at intervals to obtain the growth curve of the strain WYJY-01 (Fig. 1C). According to the growth curve, the strain WYJY-01 belongs to the lipophilic microorganism. The strain WYJY-01 was incubated at 37 °C for about 40 h in NB medium supplemented with 1% Tween 80 at a plateau stage.

### Pathogenic phenotype of *C*. *pyruviciproducens* strain WYJY-01

**Fig. 3:**
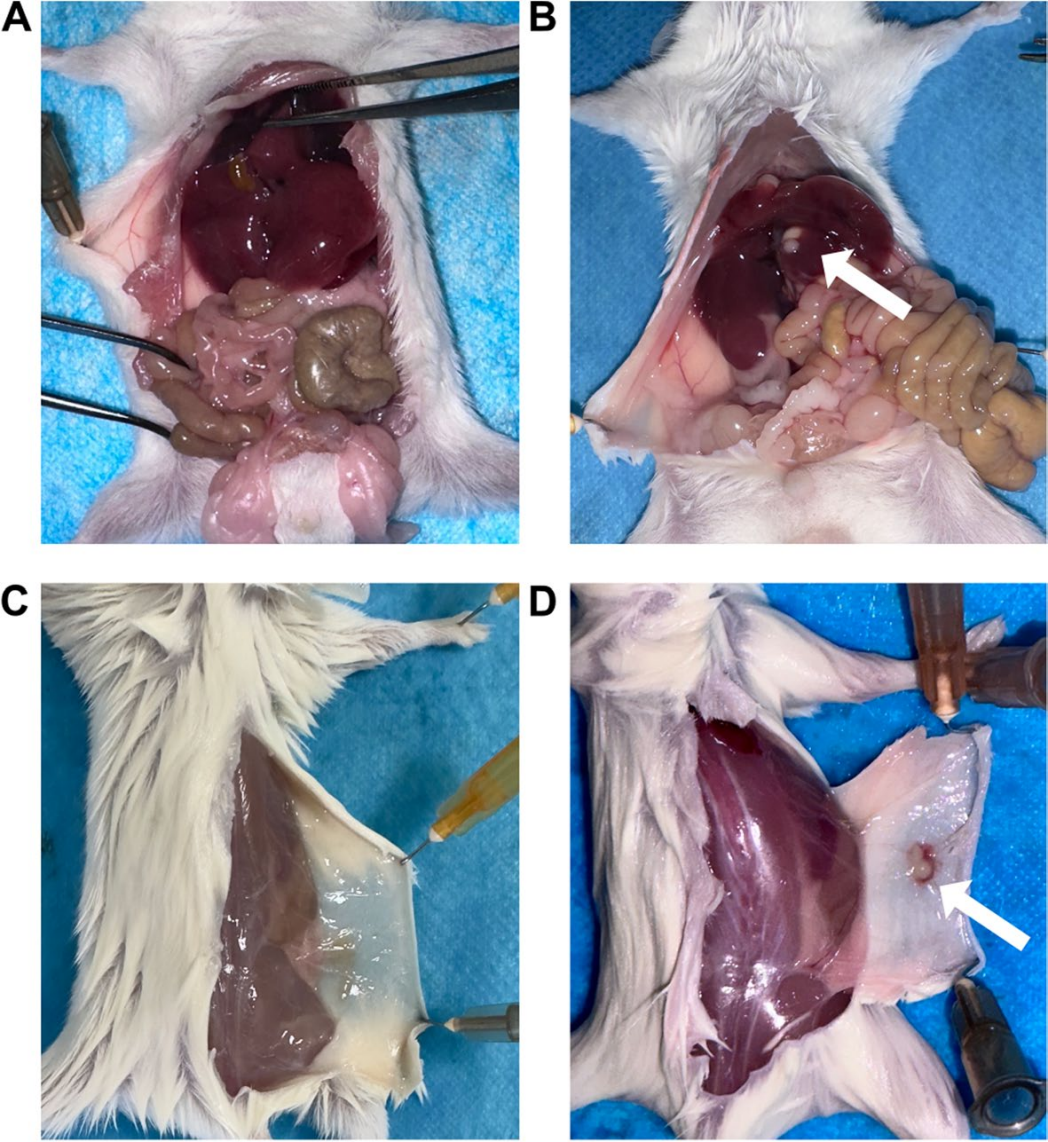
The macroscopic changes in the livers and skins of BALB/c mice infected with *C. pyruviciproducens* strain WYJY-01 in different ways

The macroscopic changes in the organs of BALB/c mice in each group were observed after being dissected. Compared with BALB/c mice injected intraperitoneally with normal saline (Fig. 3A), BALB/c mice injected intraperitoneally with the strain WYJY-01 showed scattered small abscesses on the surface of the liver (Fig. 3B). Compared with BALB/c mice injected with normal saline subcutaneously (Fig. 3C), BALB/c mice injected with the strain WYJY-01 subcutaneously had abscess formation at the injection site (Fig. 3D).

**Fig. 4:**
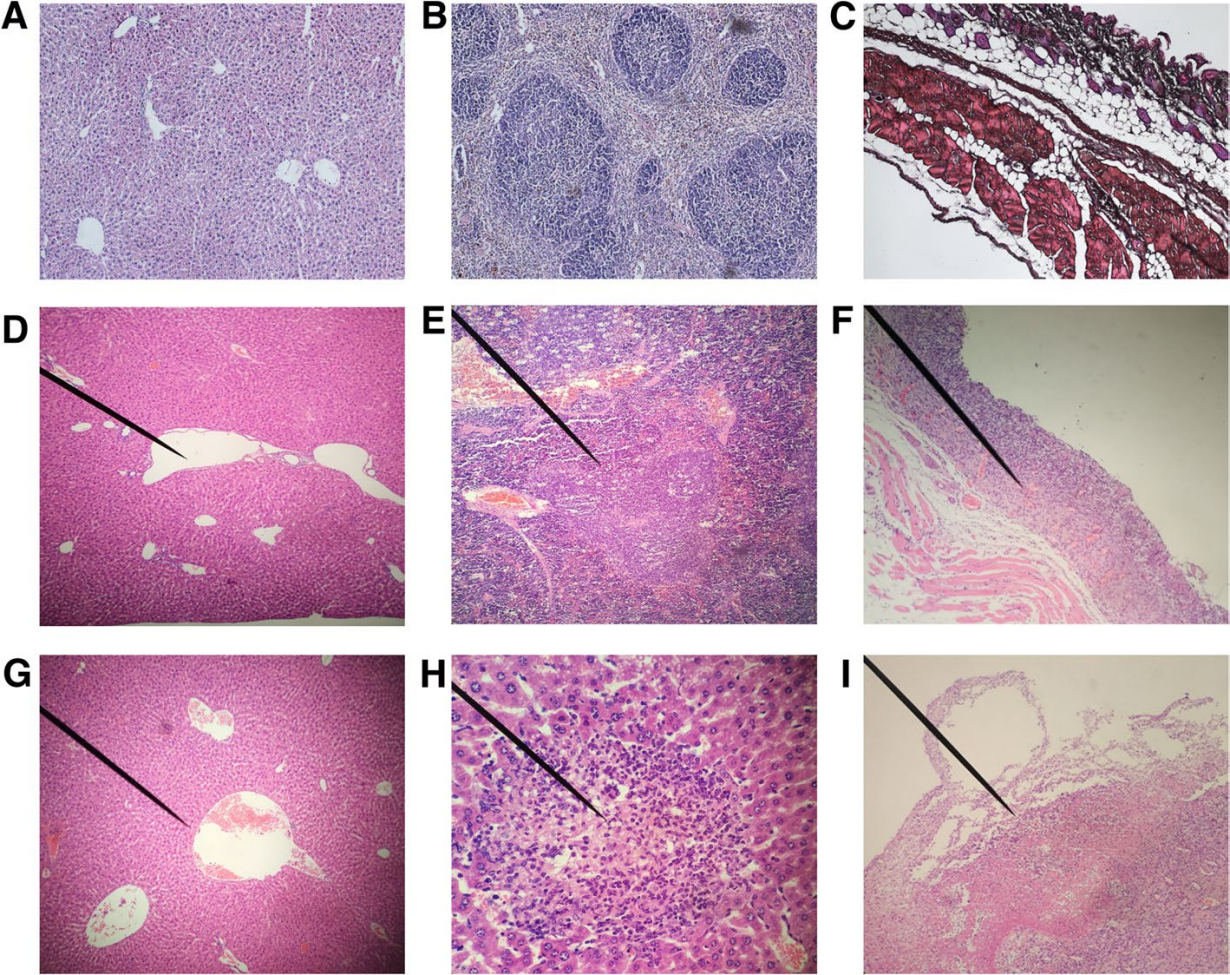
Pathological changes of BALB/c mice infected with *C. pyruviciproducens* strain WYJY-01 in different ways. **A** HE staining of normal liver tissue. **B** HE staining of normal spleen tissue. **C** HE staining of normal skin tissue. **D** Cysts of different sizes appear in the portal area of ​​the liver. **E** Reactive hyperplasia of splenic bodies. **F** Inflammatory infiltration of the subcutaneous tissue. **G** Central vein dilation in part of the liver. **H** Infiltration of lymphocytes and neutrophils in liver tissue. **I** Inflammatory necrosis, fibrinoid exudate, and granulation tissue hyperplasia in the subcutaneous tissue

The liver, spleen, and skin of normal and diseased BALB/c mice were taken and paraffin sectioned. After hematoxylin–eosin (HE) staining, the pathological changes in the tissue structure were observed under the light microscope (Fig. 4). The liver portal area of BALB/c mice injected intraperitoneally with the strain WYJY-01 showed cysts of different sizes and irregular shapes, suggesting cyst formation in the portal area (Fig. 4D) and partial central vein dilatation (Fig. 4G); Lymphocyte and neutrophil infiltration can be seen in the liver (Fig. 4H). The spleen of BALB/c mice injected intraperitoneally with the strain WYJY-01 showed reactive proliferation of splenic corpuscles (Fig. 4E). The subcutaneous tissue of BALB/c mice injected subcutaneously with the strain WYJY-01 showed inflammatory infiltration (Fig. 4F), inflammatory necrosis, cellulose-like exudation, and granulation tissue hyperplasia (Fig. 4I).

After the subcutaneous abscess drainage fluid of BALB/c mice that were subcutaneously injected with the strain WYJY-01 was smeared directly, heated, and fixed. Gram-positive and rod-shaped bacteria were seen after Gram staining. The abscess drainage fluid was cultured on Columbia blood plate at 37 °C for 48 h. The colonies isolated and cultured are round, smooth, central bulge, and transparent (Additional file 2: Fig. S1A). Some of the isolated colonies in the blood plate were Gram-stained. Gram-positive and rod-shaped bacteria (Additional file 2: Fig. S1B) were observed under the light microscope, and their morphology was the same as that of the strain WYJY-01. Moreover, the 16S rRNA gene sequence of the isolated colonies from the subcutaneous abscess drainage fluid is consistent with the 16S rRNA gene sequence of the strain WYJY-01. This proves that the colonies recovered as *C. pyruviciproducens* strain WYJY-01.

### Genomic characteristics of *C*. *pyruviciproducens* strain WYJY-01

The genome of the strain WYJY-01 was extracted and electrophoresed through agarose gel (Additional file 3: Fig. S2A). Based on the Oxford Nanopore technology platform, the raw sequencing results of the strain WYJY-01 genome showed that the Reads N50 length was 18,025 bp (Additional file 3: Fig. S2B), and the MeanQual was 10.7. Therefore, the sequencing result of the strain WYJY-01 genome was high quality.

The assembled contig sequence was aligned with the Nucleotide database to determine the chromosome type. The assembly generated a single circular chromosome with total size of 2,881,050 bp, and the GC content was 60.76%. The GC content of the strain ATCC BAA-1742^ T^ genome was 61.1% (BioProject ID PRJNA78965). The GC content of the strain UMB0763 genome was 60.8% (BioProject ID PRJNA316969). It can be seen that the difference in GC content among the three *C. pyruviciproducens* species was less than 1%. The results based on the GC content also supplemented the identification of the species of the strain WYJY-01.

**Fig. 5:**
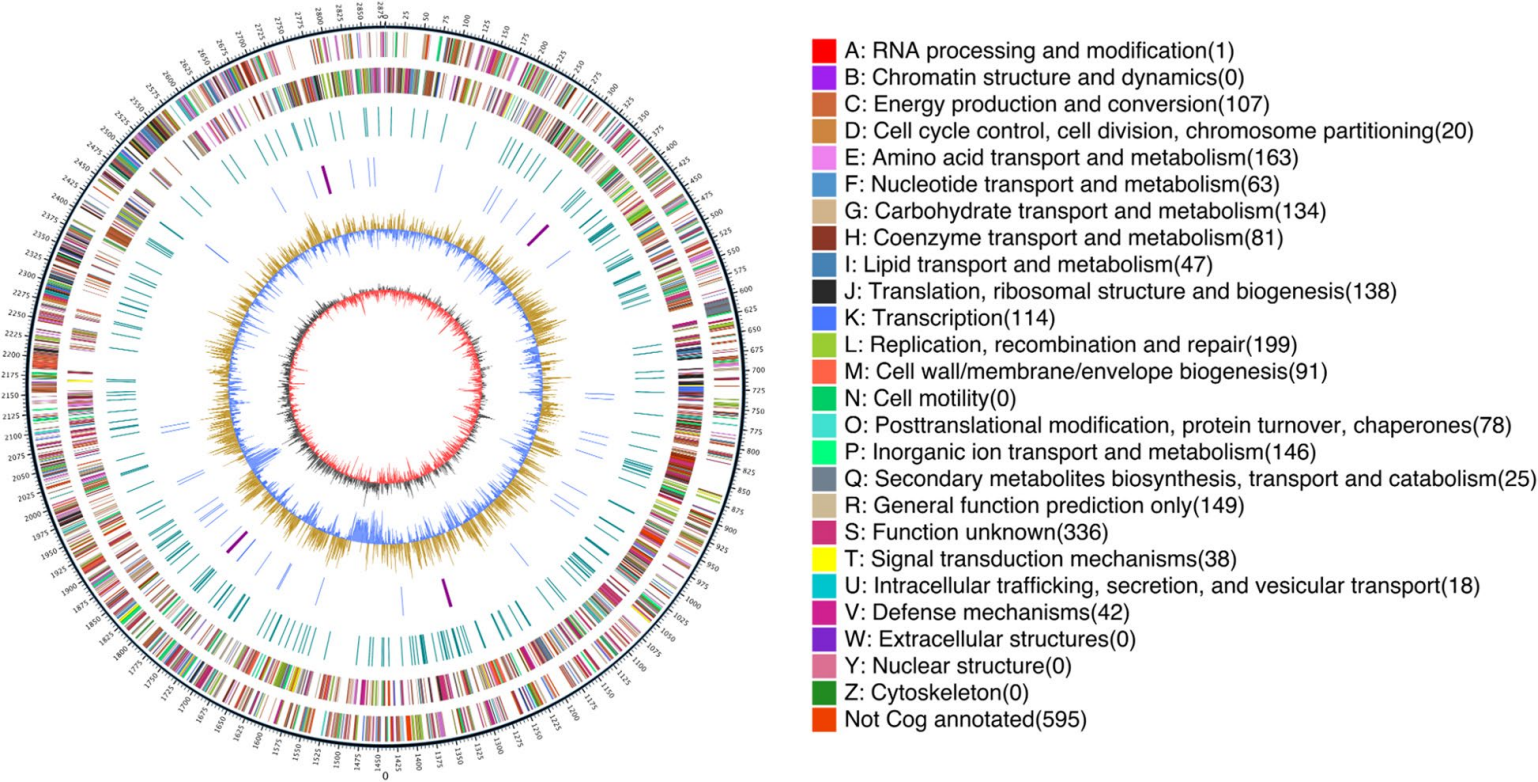
Circular genome map of *C. pyruviciproducens* strain WYJY-01. The outermost circle is the indication of genome size, each scale is 5 kb; the second and third circles are genes on the positive and negative strands of the genome, respectively, and different colors represent different COG functional classifications; the fourth circle is repetition Sequence; the fifth circle is tRNA and rRNA, blue is tRNA, and purple is rRNA; the sixth circle is GC content, the light yellow part indicates that the GC content in this region is higher than the average GC content of the genome, and the higher the peak, the higher the average GC content. The greater the difference, the blue part indicates that the GC content of the region is lower than the average GC content of the genome; the innermost circle is the GC-skew, dark gray represents the region with G content greater than C, and red represents the region with C content greater than G

The genome of the strain WYJY-01 was compared with the most similar sequence in the Nr database by BLAST. Thus, the species distribution of the matched sequence was calculated (Additional file 4: Fig. S3A). The statistical chart showed that 85.71% of the sequences in the genome of the strain WYJY-01 were most similar to *C. pyruviciproducens*, and 4.34% of the sequences were similar to *C.glucuronolyticum*. Based on the genome components predicted by the database and the gene COG function classification predicted by the eggNOG database (Additional file 4: Fig. S3B), the location of annotated loci between the genome components of the strain WYJY-01 was presented by Circos v0.66 (Fig. 5).

The ANI value based on the strain WYJY-01 and ATCC BAA-1742^ T^ was 97.44%. And the ANI value based on the strain WYJY-01 and UMB0763 was 98.56%. The dDDH value based on the strain WYJY-01 and ATCC BAA-1742^ T^ was 76.70%. And the dDDH value based on the strain WYJY-01 and UMB0763 was 87.00%. Venn diagram of gene families of the strain WYJY-01, ATCC BAA-1742^ T^, and UMB0763 genome showed (Additional file 5: Fig. S4) that there were 19 unique gene families in the genome of the strain WYJY-01, and 128 gene families different from strain ATCC BAA-1742^ T^ genome. Furthermore, the number of unique genes of the strain WYJY-01 was 314, and the number of differential genes different from strain ATCC BAA-1742^ T^ was 441.

### Virulence factors and antimicrobial resistance genes

By searching the Comprehensive Antibiotic Resistance Database (CARD) using BLSAT, the predicted resistance genes in the genome of the strain WYJY-01 were *ermX, aph(3')-Ia,aph(3″)-Ib,aph(6)-Id, cmx*. ErmX is an rRNA methyltransferase that protected the ribosome from inactivation due to antibiotic binding. Therefore, ErmX made bacteria resistant to macrolide antibiotics, lincosamide antibiotics, and streptogramin antibiotics by changing the target of antibiotics. APH(3')-Ia, APH(3″)-Ib, APH(6)-Id were aminoglycoside phosphotransferases, which can enable bacteria to acquire resistance against aminoglycoside antibiotics by inactivating antibiotics. Cmx was a chloramphenicol exporter, which can make bacteria resistant to phenicol antibiotics and chloramphenicol by exuding antibiotics.

By searching the Virulence Factor Database (VFDB) using BLAST, it was predicted that the genes in the genome of the strain WYJY-01 might be involved in encoding the virulence factors, including capsule, intercellular adhesion proteins, hemolysin, type III secretion system (TTSS), type IV secretion system, type VI secretion system, type VII secretion system, rhamnolipid, mucoid exopolysaccharide, SpaA-type pilus, polar flagella, biofilm, adhesin, metal ions transporter (mainly iron ions), P60 extracellular protein, etc.

Horizontal gene transfer is the exchange of genetic information between different species of bacteria through the mobile components. Mobile components can carry virulence factor genes and resistance genes, including genomic islands, prophages, insertion sequences, integrons, transposons, plasmids, etc. [16, 17].

**Fig. 6:**
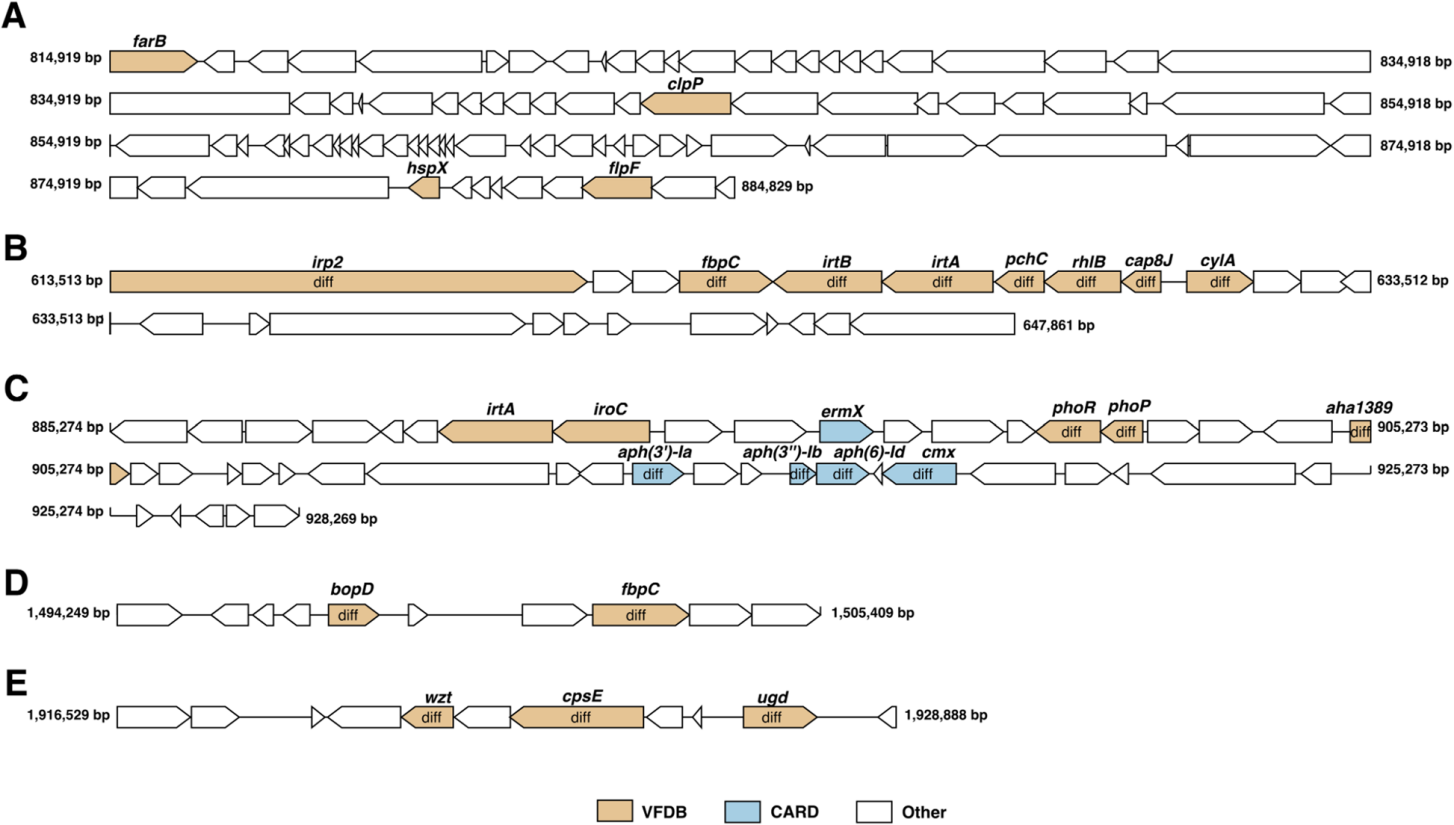
Horizontal gene transfer of virulence factor genes and resistance genes in the genome of *C. pyruviciproducens* strain WYJY-01. **A** Virulence factor genes in prophage regions in the genome of the strain WYJY-01. **B**-**E** Resistance genes and virulence factor genes in genomic islands in the genome of the strain WYJY-01. Diff, expressed as the differential gene between the strain WYJY-01 and ATCC BAA-1742^ T^

The prophage region of the genome of the strain WYJY-01 predicted by PhiSpy v2.3 was 69,911 bp in length (starting at 814,919 bp and ending at 884,829 bp). Combined with the VFDB prediction results, the virulence factor genes in the prophage region of the genome include *farB*, *clpP*, *hspX*, and *flpF* (Fig. 6A). Among them, FarB was a fatty acid efflux system protein, and it mediated the resistance to antimicrobial long-chain fatty acids. HspX stabilized cell structure during long-term survival and permitted the bacilli to survive within the low-oxygen environment of the granuloma.

There were 13 genomic islands predicted by IslandPath-DIMOB v0.1 in the genome of the strain WYJY-01. Genomic island 4 (Fig. 6B) started at 613,513 bp and ended at 647,861 bp. The virulence factor genes in this genomic island include *irp2*, *fbpC*, *irtB*, *irtA*, *pchC*, *rhlB*, *cap8J*, and *cylA*. Meanwhile, these genes were the different genes that were different from the genome of the strain ATCC BAA-1742^ T^. Genomic island 6 (Fig. 6C) started at 885,274 bp and ended at 928,269 bp. The genomic island contained virulence factor genes, including *irtA*, *iroC*, *phoR*, *phoP*, and *aha1389*. It was worth noting that the five resistance genes in the genome of the strain WYJY-01 searched by the CARD were all located on Genomic island 6. Genomic island 8 (Fig. 6D) started at 1,494,249 bp and ended at 1,505,409 bp, and Genomic island 10 (Fig. 6E) started at 1,916,529 bp and ended at 1,928,888 bp. The virulence factor genes contained in these two genomic islands included *bopD*, *fbpC*, *wzt*, *cpsE*, and *ugd*, and these genes were also differential genes from the genome of the strain ATCC BAA-1742^ T^.

**Fig. 7:**
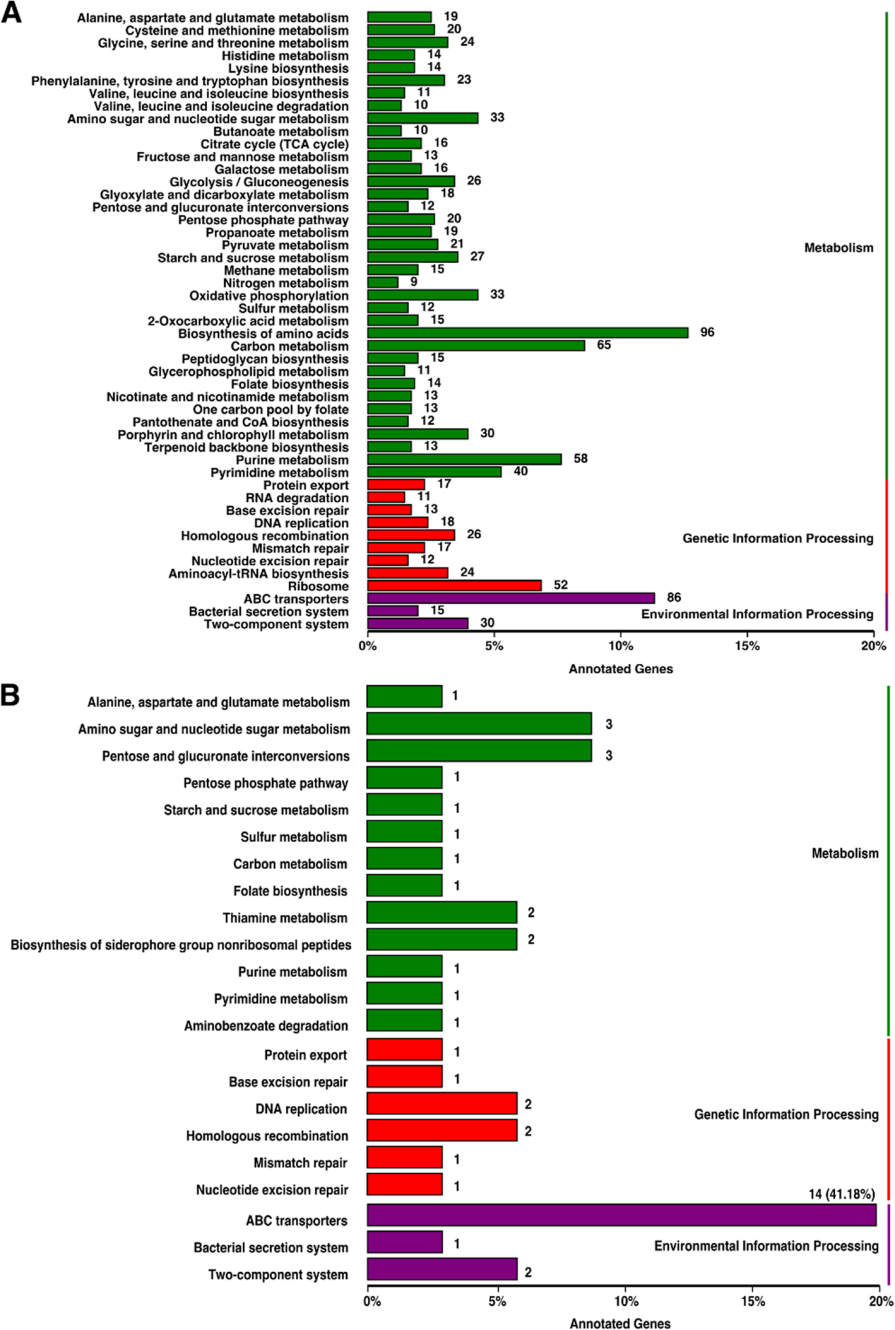
The KEGG database annotations of the genome of *C. pyruviciproducens* strain WYJY-01 and its differential genes with that of ATCC BAA-1742^ T^. **A** KEGG cluster analysis of the genome of the strain WYJY-01. **B** KEGG cluster analysis of differential genes between the genomes of the strain WYJY-01 and ATCC BAA-1742^ T^

The results of gene function annotated by the KEGG database (Fig. 7) showed the functions of the differential genes between the strain WYJY-01 and the strain ATCC BAA-1742^ T^ were mainly clustered in environmental information processing (Fig. 7B). The differential genes clustered into ABC transporter and predicted by VFDB included *fbpC* on Genomic island 4, *fbpC* on Genomic island 8, and *wzt* on Genomic island 10, *shuU*, and *fepC*. One of the differential genes clustered into the two-component system was the *phoR* of Genomic island 6. The results of gene function annotated by the GO database showed (Additional file 6: Fig. S5) that the proportion of differential genes in the two categories of the extracellular region and electron carrier activity in each functional category was significantly higher than that of the genome of the strain WYJY-01.

### Secondary metabolite gene clusters

**Fig. 8:**
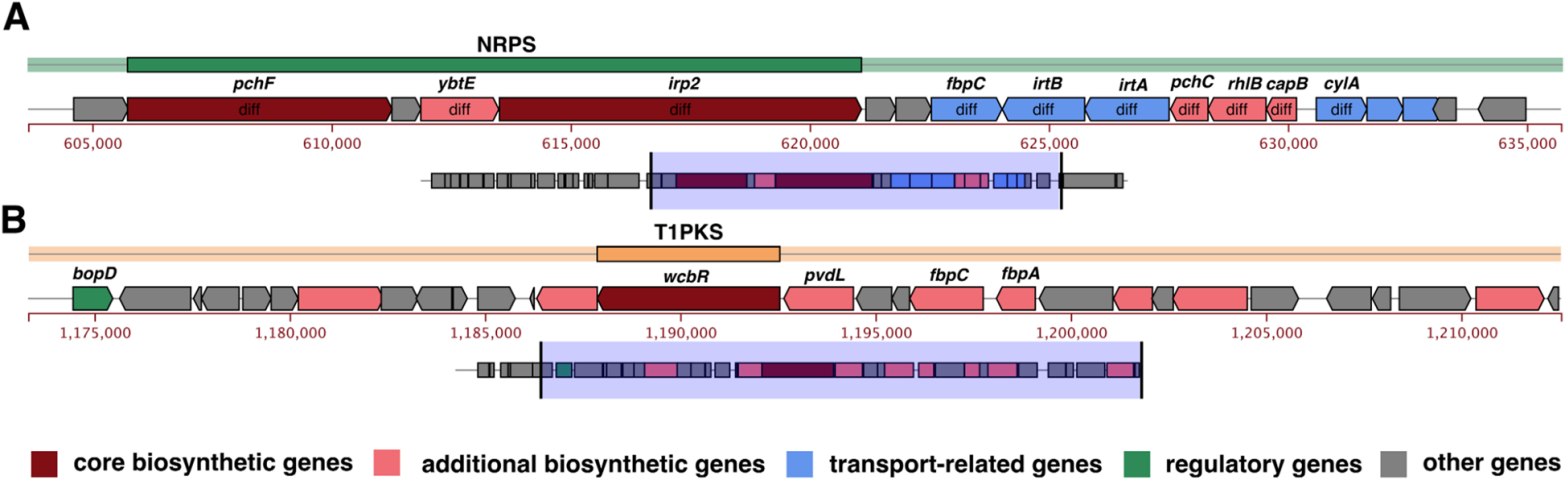
Secondary metabolite gene clusters in the genome of *C. pyruviciproducens* strain WYJY-01. **A** Prediction of non-ribosomal polypeptide (NRPS) gene clusters in the genome of the strain WYJY-01. **B** Prediction of the polyketide (T1PKS) gene cluster in the genome of the strain WYJY-01. Diff, expressed as the differential gene between the strain WYJY-01 and ATCC BAA-1742^ T^

The secondary metabolite gene clusters of the strain WYJY-01 predicted by antiSMASH v5.0.0 consists of two regions. The non-ribosomal polypeptides (NRPS) gene cluster (Fig. 8A) in the genome started at 585,737 bp and ended at 641,084 bp, and the secondary metabolites were roughly predicted as (cys) + (cys—cys), peptide bonds linked three Cysteines. The polyketides (T1PKS) gene cluster (Fig. 8B) in the genome started at 1,167,876 bp and ended at 1,212,552 bp, and the secondary metabolite was roughly predicted to be pyruvic acid (CH3COCOOH). It was worth noting that the core structure region of the NRPS gene cluster had overlapping genes with Genomic island 4 (Fig. 6B), and most of the genes in the core structure region were the differential genes from the genomic of the strain ATCC BAA-1742^ T^.

## Discussion

*C. pyruviciproducens* is a lipogenic *Corynebacterium* capable of producing pyruvate, which was named *C. pyruviciproducens* strain ATCC BAA-1742^ T^ in 2006 by Tong J [10]. A clinical isolate of *C. pyruviciproducens* involved in this reaearch was named *C. pyruviciproducens* strain WYJY-01. The colony morphology of the two *C. pyruviciproducens* strains was consistent, and the morphology was consistent under the microscope after Gram staining, and both were lipophilic bacteria. In addition, the GC content and ANI and DDH analysis of the genomes of both strains indicated that they were of the same species [18]. However, there were some differences in other general microbiological phenotypes of the two strains, such as bacterial biochemical reactions, antimicrobial susceptibility tests, etc. It is worth noting that, in our research on animal models with different infection routes, we found that under the guidance of Koch’s postulates [19–21], the strain WYJY-01 may be pathogenic. The strain WYJY-01 can infect BALB/c mice through intraperitoneal and subcutaneous routes, and cause significant organic changes in BALB/c mice. As *C. pyruviciproducens* has been discovered for a short time and there are few relevant studies, these data are of great significance for public health monitoring and provide a reference for the phenotypic identification of this bacterium in future clinical work.

At present, studies on the genomic characteristics of *C. pyruviciproducens* are scarce. We extracted the genomic data of *C. pyruviciproducens* strain ATCC BAA-1742^ T^ and UMB0763 from the NCBI Genome database. These two *C. pyruviciproducens* strains are clinical isolates. However, the microbiological phenotype description of strain UMB0763 is not clear [22]. Therefore, we focus on the results of the genomic analysis of *C. pyruviciproducens* strain WYJY-01 and ATCC BAA-1742^ T^.

Horizontal gene transfer can lead to DNA exchange between different species of bacteria, thereby enhancing their adaptability to the environment, and is one of the important pathways for bacterial evolution. Genomic islands are very active carriers in horizontal gene transfer, and play a crucial role in the plasticity and evolutionary evolution of bacteria [23]. Genomic islands can carry virulence factor genes, resistance genes, metabolic regulation genes, etc., which can lead to the production of pathogenic multi-drug resistant bacteria [24, 25].

Firstly, through CARD resistance gene prediction and comparative genomics analysis, it was concluded that the resistance phenotype and genotype of the strain WYJY-01 against aminoglycoside antibiotic, macrolide antibiotic, and lincosamide antibiotic are consistent. However, the genotype associated with the drug resistance phenotype of the strain WYJY-01 against quinolones antibiotic was not predicted. Moreover, the genotype of the strain WYJY-01 resistant to chloramphenicol was predicted. Combined with the genomic islands prediction results of the strain WYJY-01 genome, it can be seen that five resistance genes of the strain WYJY-01 were located in Genomic island 6 (Fig. 6C). Therefore, Genomic island 6 is the resistance island of the strain WYJY-01, and it may play a decisive role in the process of bacterial resistance.

Secondly, pathogenic bacteria have gone through many processes from adsorption into host cells to successful infection, such as adsorption, colonization, escape from host immune response, local disease, and other stages. According to the prediction results of virulence factors of VFDB, the genome of the strain WYJY-01 contains genes related to virulence factors at various stages of bacterial infection of the host. Combined with the genomic islands prediction results of the strain WYJY-01 genome, it can be seen that the virulence factor genes in Genomic island 4 (Fig. 6B), such as *irp2* [26], *fbpC* [27], *irtA*, *irtB* [28], *pchC* [29, 30], were mainly involved in the biological process of bacterial iron ion transport. This process plays an essential role in regulating the local pathogenic effect stage of bacteria, because the ability of pathogenic bacteria to compete with the host for iron ions plays an important role in bacterial survival and virulence [31]. Virulence factor genes in Genomic island 10 (Fig. 6E), such as *cpsE* [32] and *ugd* [33], were mainly involved in the formation process of the capsule, assisting in evading the host immune system by protecting bacteria from opsonophagocytosis and serum killing. Finally, it was predicted through the KEGG database that the genome of the strain WYJY-01 and the differential genes of the strain WYJY-01 and ATCC BAA-1742^ T^ were mainly clustered into the ABC transporters module of environmental information processing. Further research found that the differential genes clustered to ABC transporters were mainly involved in the transport of iron ions [28], such as *fbpC*, *wzt*, *shuU*, *fep*C, etc. This further proved that the strain WYJY-01 showed a possible molecular mechanism at the gene level of the pathogenic phenotype.

Furthermore, we also predicted the gene cluster of the secondary metabolite of the strain WYJY-01. Through the analysis of the T1PKS database, *C. pyruviciproducens* had the core gene cluster of pyruvate (Fig. 8B), and the gene sequence of the T1PKS gene cluster also appeared in the genome of the strain ATCC BAA-1742^ T^. The core biological gene of the T1PKS gene cluster was also annotated as *wcbR* by VFDB, encoding capsular polysaccharide biosynthesis fatty acid synthase, which was the critical virulence determinant [34]. Through the NRPS database, we predicted that the strain WYJY-01 might metabolize into a compound consisting of three cysteines linked by a peptide bond. At the same time, the NRPS gene cluster overlaps with Genomic island 4 (Fig. 6B). This showed that Genomic island 4 was not only the core pathogenicity island of the strain WYJY-01, but also the metabolism island of the strain WYJY-01.

## Conclusion

In general, this study supplemented the microbiological phenotypic characteristics of *C. pyruviciproducens*, and especially explored its pathogenic phenotype. Through whole-genome sequence analysis, this study revealed the possible molecular mechanism of the pathogenicity of this clinical isolate at the gene level, and also provided a basis for further research on the molecular basis of the relevant microbiological phenotypic characteristics of this species. This study expanded the comprehension for identifying, analyzing, and treating *C. pyruviciproducens* in clinical.

## Methods

### Identification of clinical isolates and general microbiological phenotypes

The clinical isolates of *C. pyruviciproducens* involved in this study were from a female patient with sebaceous gland cysts on the skin near the sternum in the Affiliated Hospital of Weifang Medical University. The Medical Ethics Committee of Weifang Medical University approved the study (No. 2022YX65) and waived the informed consent requirement because this study used only bacterial isolate and did not have any negative impact on patients. After the abscess was cut and drained, the clinical isolates were obtained, and the pus samples were isolated and cultured in our hospital. First, the isolate was identified and analyzed by VITEK 2 Compact full-automatic microbial analyzer [35]. Then the isolated bacteria were observed under the light microscope through Gram staining. Due to the inconsistency between the theoretical bacterial morphology predicted by the analyzer and the observed bacterial morphology under the light microscope after Gram staining, the isolate was identified by 16S rRNA and *rpoB* gene sequencing, and was named *C. pyruviciproducens* strain WYJY-01 [36, 37]. The 16S rRNA gene sequence of *C. pyruviciproducens* strain WYJY-01 was uploaded to the GenBank database. Preliminary sequence analysis of the 16S rRNA gene was conducted using the NCBI database, and strains with high homology in the NCBI database were selected for phylogenetic tree analysis. The phylogenetic tree was constructed by using Mega10.0 and the Neighbor-Joining method. The percentage of replicate trees in which the associated taxa clustered together in the bootstrap test (1000 replicates) are shown next to the branches.

Next, the drug resistance phenotype of the isolate was determined by the Etest, a reference method for testing antimicrobial susceptibility to diverse drugs in accordance with CLSI guidelines [15]. In addition, the isolate was cultured in NB medium supplemented with 1% Tween 80, which further verified its lipid requirement and measured its growth curve [38, 39].

### Study on pathogenic phenotypes based on Koch’s postulates

All experiments involving mice were approved by the Institutional Animal Care and Use Committee of Weifang Medical University (No. 2022SDL162). SPF grade experimental mice were BALB/c, male, weighing about 25–30 g, purchased from Qingdao Da Ren Fucheng Livestock Co., Ltd. *C. pyruviciproducens* strain WYJY-01 was resuscitated in Columbia blood agar medium. The colony was scraped and resuspended in normal saline, and adjusted to about 3 × 10^9^ CFU/mL. Twenty-four BALB/c mice were randomly divided into four groups. In the first group, 6 mice were intraperitoneally injected with 1 mL of bacterial fluid. In the second group, 6 mice were injected with 100 μL of bacterial fluid subcutaneously. The third group was the control group. 6 mice were intraperitoneally injected with 1 mL sterile normal saline. The fourth group was the control group. 6 mice were subcutaneously injected with 100 μL sterile normal saline. After 3 days of injection, 6 mice in each experimental group were dissected, and taken out the relevant diseased organs to make ordinary paraffin sections. After HE staining, the changes in tissue structure were observed. The subcutaneous abscess drainage fluid of the infected model was isolated and cultured again. The isolated strains were identified.

### Whole genome sequencing and assembly

*C. pyruviciproducens* strain WYJY-01 stored at -80 °C was resuscitated on Columbia blood agar medium and grew at 37 °C for 48 h. The fresh isolates were resuspended in molecular biology-grade water without nuclease. According to instructions, the collected *C. pyruviciproducens* strain WYJY-01 was extracted with a QIAGEN Genomic-tip DNA kit. The harvested DNA was electrophoresed through agarose gel and quantified by Qubit assays (Invitrogen). The concentration of the extracted genome was 70.2 ng/μL. OD_260/280_ of the gemome was 1.87, and OD_260/230_ of the gemome was 2.04. Whole genome sequencing was done by Oxford Nanopore Technologies DNA sequencing platform (Biomarker Technologies, Beijing, China). The reads after the quality control were assembled by Canu v1.5 [40]. Finally, we polished the assemblies using Pilon v1.22 [41]. The assembled *C. pyruviciproducens* strain WYJY-01 genome was uploaded to the NCBI database.

### Genomic analysis

Prodigal v2.6.3 [42] was used to predict coding sequences (CDSs) in the genome of the strain. RepeatMasker v4.0.5 [43] was used to search for repetitive sequences. Rfam v12.0 [44], and tRNAscan-SE v1.3.1 [45] were used to predict tRNA, and other ncRNAs. And genome information obtained by assembly and prediction, such as information on tRNAs, rRNAs, repeat sequences, GC contents, and gene functions, was used to draw a circular genome map with the software Circos v0.66 [46].

The genomic islands and the prophage were predicted by IslandPath-DIMOB v0.1 [47] and PhiSpy v2.3 [48]. The gene sequence was compared with eggNOG [49], KEGG [50], Nr, and other functional databases by BLAST v2.2.29 to provide the functional annotation. Based on the comparison results of the Nr database, the gene sequence was annotated by Blast2GO v2.5 [51] for the functions of the GO database. The protein sequences of the strain WYJY-01 were blasted by the CARD [52] and VFDB [53], and the corresponding annotation results were obtained. The secondary metabolite gene cluster was predicted by antiSMASH v5.0.0 [54].

The Average Nucleotide Identity (ANI) and DNA–DNA hybridization (dDDH) of the genomic sequences of *C. pyruviciproducens* strain WYJY-01, ATCC BAA-1742^ T^, and UMB0763 were analyzed by fastANIv1.33 [55] and GGDC v3.0 [56]. The protein sequences of *C. pyruviciproducens* strain WYJY-01, ATCC BAA-1742^ T^, and UMB0763 were classified by the OrthoMCL [57]. Then, gene families were analyzed, including the specific gene family of the strain WYJY-01, the gene family shared by the three strains, and the gene family with a single copy of each strain. The differential genes of the strain WYJY-01 between the strain ATCC BAA-1742^ T^ were annotated by KEGG metabolic pathway and GO annotation.

### Supplementary Information

**Additional file 1:** **Table S1.** Biochemical traits of *C*. *pyruviciproducens* isolates.

**Additional file** **2: Fig. S1.** Subcutaneous abscesses of BALB/c mice after subcutaneous infection with *C*. *pyruviciproducens* strain WYJY-01 were re-isolated, cultured, and identified by Gram staining.

**Additional file 3: Fig. S2.** Quality control of whole genome extraction and sequencing data of *C*. *pyruviciproducens* strain WYJY-01.

**Additional file 4: Fig. S3.** Genome annotation of *C*. *pyruviciproducens* strain WYJY-01.

**Additional file 5: Fig. S4.** Venn diagram based on gene families clustered from the Pfam database of the genomes of three strains of *C*. *pyruviciproducens* strain WYJY-01, ATCC BAA-1742T, and UMB0763.

**Additional file 6: Fig. S5.** The GO database annotations of the genome of *C*. *pyruviciproducens* strain WYJY-01 and its differential genes with that of ATCC BAA-1742T.

## Data Availability

All data generated or analyzed during this study are included in this published article and its supplementary information files. The GenBank accession numbers for the whole-genome and 16S rRNA gene sequences of *C. pyruviciproducens* strain WYJY-01 are SRX14997086 and OP799734.1, respectively.
