# Supplementary material for: Genomic and phenotypic analysis of a novel clinical isolate of Corynebacterium pyruviciproducens
Source: BMC Microbiol. 2023 Dec 6;23:385. doi: 10.1186/s12866-023-03075-6 (PMC10699042; doi:10.1186/s12866-023-03075-6)
Supplement: Supplementary file 1 — Additional file 1: Table S1. Biochemical traits of C. pyruviciproducens isolates. [file 12866_2023_3075_MOESM1_ESM.pdf]

Genomic and phenotypic analysis of a novel clinical isolate of *Corynebacterium pyruviciproducens*

Jiaqi Wang[1,2], Jiajia Feng[3], Wei Jia[4], Tingxun Yuan[1,2], Xinyu He[1,2], Qianqian Wu[5], Fujun Peng[6], Wei Gao[7], Zhongfa Yang[6], Yuanyong Tao[5*], Qian Li [1,2*]

[1]School of Medical Laboratory, Weifang Medical University, Weifang, Shandong 261053, PR China

[2]Engineering Research Institute of Precision Medicine Innovation and Transformation of Infections Diseases, Weifang Medical University, Weifang, Shandong 261053, PR China

[3]Clinical Laboratory, Weifang Maternal and Child Health Care Hospital, Weifang, Shandong 261011, PR China

[4]Clinical Laboratory, Weifang People's Hospital, Weifang, Shandong 261000, PR China

[5]Clinical Laboratory, the Affiliated Hospital of Weifang Medical University, Weifang 261031, PR China

[6]School of Basic Medical Sciences, Weifang Medical University, Weifang, China

[7]Key Lab for Immunology in Universities of Shandong Province, Weifang Medical University, Weifang, Shandong 261053, PR China

[*]Corresponding authors:

Yuanyong Tao, Email: taoyuanyong@163.com.

Qian Li, Email: liqian@wfmc.edu.cn.

Table S1 Biochemical traits of *C. pyruviciproducens* isolates

Biochemical trait	Results for:	
	strain ATCC BAA-1742^T	Strain WYJY-01
CAMP reaction	-	+
Reduction of nitrates	-	+
Catalase	+	+
Production of:		
β-Glucuronidase	+	+
Urease	-	-
α-Glucosidase	-	-
β- Glucosidase	-	-
N-acetyl-β-glucosaminidase	-	-
Fermentation of:		
D-Ribose	+	-
D-Xylose	+	-
D-Glucose	+	-
Maltose	+	-
Sucrose	+	-
Fructose	+	ND[a]
D-Mannitol	-	-

[a]ND= Not done.
