# Supplementary material for: Genomic and phenotypic analysis of a novel clinical isolate of Corynebacterium pyruviciproducens
Source: BMC Microbiol. 2023 Dec 6;23:385. doi: 10.1186/s12866-023-03075-6 (PMC10699042; doi:10.1186/s12866-023-03075-6)
Supplement: Supplementary file 2 — Additional file 2: Fig. S1. Subcutaneous abscesses of BALB/c mice after subcutaneous infection with C. pyruviciproducens strain WYJY-01 were re-isolated, cultured, and identified by Gram staining. [file 12866_2023_3075_MOESM2_ESM.pdf]

Genomic and phenotypic analysis of a novel clinical isolate of *Corynebacterium pyruviciproducens*

Jiaqi Wang[1,2], Jiajia Feng[3], Wei Jia[4], Tingxun Yuan[1,2], Xinyu He[1,2], Qianqian Wu[5], Fujun Peng[6], Wei Gao[7], Zhongfa Yang[6], Yuanyong Tao[51], Qian Li [1,2*]

[1]School of Medical Laboratory, Weifang Medical University, Weifang, Shandong 261053, PR China

[2]Engineering Research Institute of Precision Medicine Innovation and Transformation of Infections Diseases, Weifang Medical University, Weifang, Shandong 261053, PR China

[3]Clinical Laboratory, Weifang Maternal and Child Health Care Hospital, Weifang, Shandong 261011, PR China

[4]Clinical Laboratory, Weifang People's Hospital, Weifang, Shandong 261000, PR China

[5]Clinical Laboratory, the Affiliated Hospital of Weifang Medical University, Weifang 261031, PR China

[6]School of Basic Medical Sciences, Weifang Medical University, Weifang, China

[7]Key Lab for Immunology in Universities of Shandong Province, Weifang Medical University, Weifang, Shandong 261053, PR China

[*]Corresponding authors:

Yuanyong Tao, Email: taoyuanyong@163.com.

Qian Li, Email: liqian@wfmc.edu.cn.

Supplementary figure 1

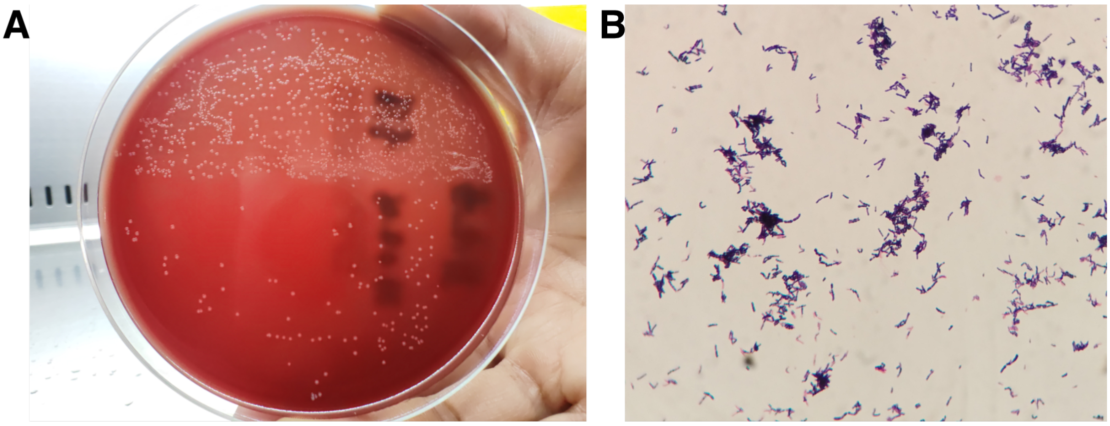

Fig. S1. Subcutaneous abscesses of BALB/c mice after subcutaneous infection with *C. pyruviciproducens* strain WYJY-01 were re-isolated, cultured, and identified by Gram staining. **(A)** Colony morphology after subcutaneous abscess pus was inoculated on Columbia blood plate and incubated at 37°C for 48 h. **(B)** The morphological characteristics of the direct smear of pus from the subcutaneous abscess under the light microscope after Gram staining.
