# Supplementary material for: Genomic and phenotypic analysis of a novel clinical isolate of Corynebacterium pyruviciproducens
Source: BMC Microbiol. 2023 Dec 6;23:385. doi: 10.1186/s12866-023-03075-6 (PMC10699042; doi:10.1186/s12866-023-03075-6)
Supplement: Supplementary file 3 — Additional file 3: Fig. S2. Quality control of whole genome extraction and sequencing data of C. pyruviciproducens strain WYJY-01. [file 12866_2023_3075_MOESM3_ESM.pdf]

Genomic and phenotypic analysis of a novel clinical isolate of *Corynebacterium pyruviciproducens*

Jiaqi Wang[1,2], Jiajia Feng[3], Wei Jia[4], Tingxun Yuan[1,2], Xinyu He[1,2], Qianqian Wu[5], Fujun Peng[6], Wei Gao[7], Zhongfa Yang[6], Yuanyong Tao[5*], Qian Li [1,2*]

[1]School of Medical Laboratory, Weifang Medical University, Weifang, Shandong 261053, PR China

[2]Engineering Research Institute of Precision Medicine Innovation and Transformation of Infections Diseases, Weifang Medical University, Weifang, Shandong 261053, PR China

[3]Clinical Laboratory, Weifang Maternal and Child Health Care Hospital, Weifang, Shandong 261011, PR China

[4]Clinical Laboratory, Weifang People's Hospital, Weifang, Shandong 261000, PR China

[5]Clinical Laboratory, the Affiliated Hospital of Weifang Medical University, Weifang 261031, PR China

[6]School of Basic Medical Sciences, Weifang Medical University, Weifang, China

[7]Key Lab for Immunology in Universities of Shandong Province, Weifang Medical University, Weifang, Shandong 261053, PR China

[*]Corresponding authors:

Yuanyong Tao, Email: taoyuanyong@163.com.

Qian Li, Email: liqian@wfmc.edu.cn.

Supplementary figure 2

A B

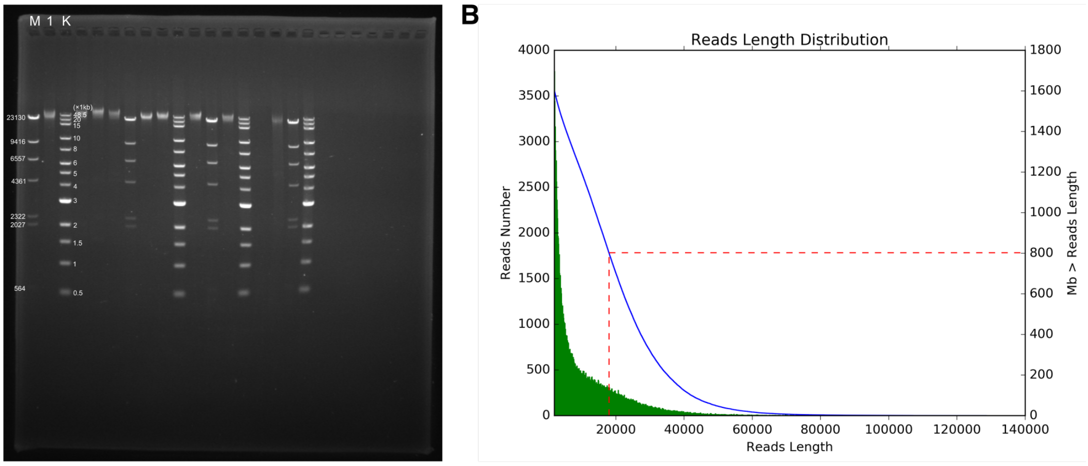

Fig. S2. Quality control of whole genome extraction and sequencing data of *C. pyruviciproducens* strain WYJY-01. **(A)** The extracted genome was detected by 0.5% agarose gel electrophoresis. Lane K: NEB Quick-Load 1 kb Extend DNA Ladder; Lane M: TAKARA λ-Hind III digest; Lane 1: Genome sample of the strain WYJY-01. **(B)** Quality control of reading length data after genome sequencing. The abscissa represents the length of reads (bp). The left ordinate represents the number of reads, corresponding to the green histogram. The right ordinate represents the total number of bases (Mb) contained in reads larger than the corresponding length, corresponding to the blue curve. Red Dashed lines indicate the N50 length of reads.
