# Supplementary material for: Genomic and phenotypic analysis of a novel clinical isolate of Corynebacterium pyruviciproducens
Source: BMC Microbiol. 2023 Dec 6;23:385. doi: 10.1186/s12866-023-03075-6 (PMC10699042; doi:10.1186/s12866-023-03075-6)
Supplement: Supplementary file 4 — Additional file 4: Fig. S3. Genome annotation of C. pyruviciproducens strain WYJY-01. [file 12866_2023_3075_MOESM4_ESM.pdf]

# Genomic and phenotypic analysis of a novel clinical isolate of *Corynebacterium pyruviciproducens*

Jiaqi Wang[1,2], Jiajia Feng[3], Wei Jia[4], Tingxun Yuan[1,2], Xinyu He[1,2], Qianqian Wu[5], Fujun Peng[6], Wei Gao[7], Zhongfa Yang[6], Yuanyong Tao[5*], Qian Li [1,2*]

[1]School of Medical Laboratory, Weifang Medical University, Weifang, Shandong 261053, PR China

[2]Engineering Research Institute of Precision Medicine Innovation and Transformation of Infections Diseases, Weifang Medical University, Weifang, Shandong 261053, PR China

[3]Clinical Laboratory, Weifang Maternal and Child Health Care Hospital, Weifang, Shandong 261011, PR China

[4]Clinical Laboratory, Weifang People's Hospital, Weifang, Shandong 261000, PR China

[5]Clinical Laboratory, the Affiliated Hospital of Weifang Medical University, Weifang 261031, PR China

[6]School of Basic Medical Sciences, Weifang Medical University, Weifang, China

[7]Key Lab for Immunology in Universities of Shandong Province, Weifang Medical University, Weifang, Shandong 261053, PR China

[*]Corresponding authors:

Yuanyong Tao, Email: taoyuanyong@163.com.

Qian Li, Email: liqian@wfmc.edu.cn.

## Supplementary figure 3

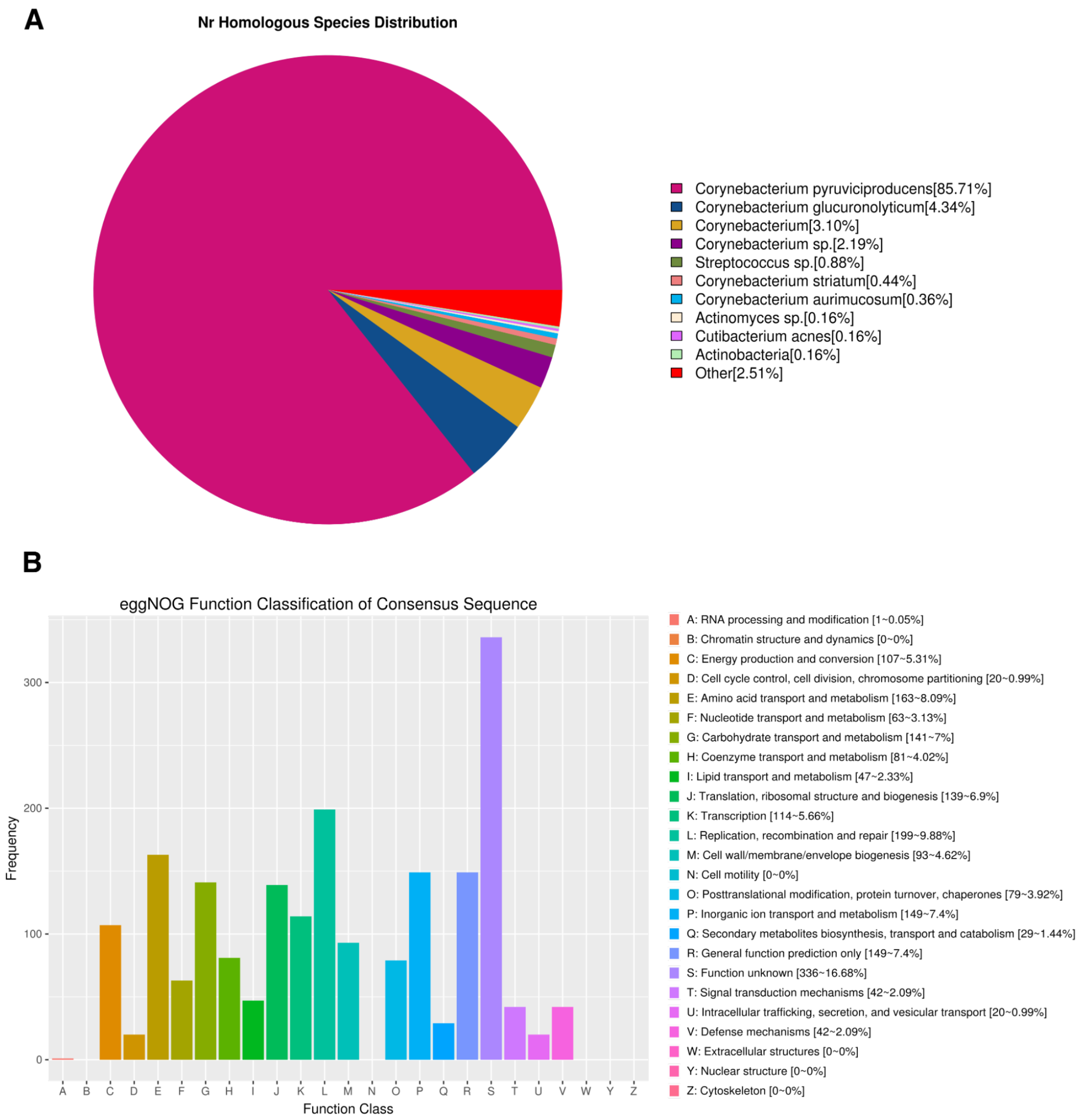


**Fig. S3.** Genome annotation of *C. pyruviciproducens* strain WYJY-01. **(A)** The species distribution map of the gene sequence alignment of strain WYJY-01 was plotted against the Nr database. **(B)** Gene functions of the genome of strain WYJY-01 were classified by alignment to the eggNOG database.
