# Supplementary material for: Genomic and phenotypic analysis of a novel clinical isolate of Corynebacterium pyruviciproducens
Source: BMC Microbiol. 2023 Dec 6;23:385. doi: 10.1186/s12866-023-03075-6 (PMC10699042; doi:10.1186/s12866-023-03075-6)
Supplement: Supplementary file 6 — Additional file 6: Fig. S5. The GO database annotations of the genome of C. pyruviciproducens strain WYJY-01 and its differential genes with that of ATCC BAA-1742T. [file 12866_2023_3075_MOESM6_ESM.pdf]

Genomic and phenotypic analysis of a novel clinical isolate of *Corynebacterium pyruviciproducens*

Jiaqi Wang[1,2], Jiajia Feng[3], Wei Jia[4], Tingxun Yuan[1,2], Xinyu He[1,2], Qianqian Wu[5], Fujun Peng[6], Wei Gao[7], Zhongfa Yang[6], Yuanyong Tao[5*], Qian Li [1,2*]

[1]School of Medical Laboratory, Weifang Medical University, Weifang, Shandong 261053, PR China

[2]Engineering Research Institute of Precision Medicine Innovation and Transformation of Infections Diseases, Weifang Medical University, Weifang, Shandong 261053, PR China

[3]Clinical Laboratory, Weifang Maternal and Child Health Care Hospital, Weifang, Shandong 261011, PR China

[4]Clinical Laboratory, Weifang People's Hospital, Weifang, Shandong 261000, PR China

[5]Clinical Laboratory, the Affiliated Hospital of Weifang Medical University, Weifang 261031, PR China

[6]School of Basic Medical Sciences, Weifang Medical University, Weifang, China

[7]Key Lab for Immunology in Universities of Shandong Province, Weifang Medical University, Weifang, Shandong 261053, PR China

[*]Corresponding authors:

Yuanyong Tao, Email: taoyuanyong@163.com.

Qian Li, Email: liqian@wfmc.edu.cn.

Supplementary figure 5

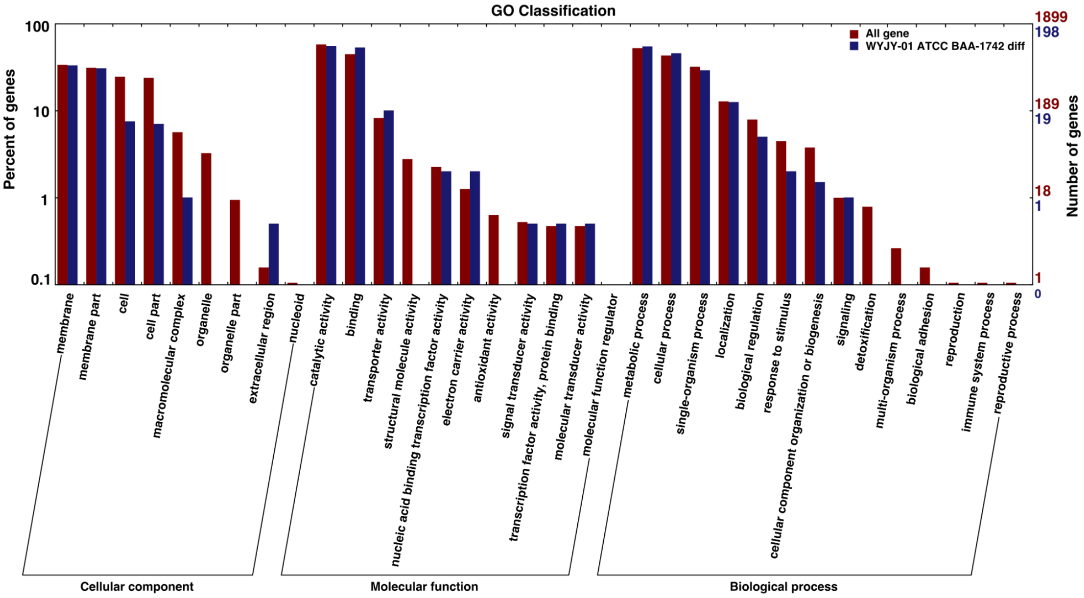

Fig. S5. The GO database annotations of the genome of *C. pyruviciproducens* strain WYJY-01 and its differential genes with that of ATCC BAA-1742^{T}.
